# How the Start into the Clinical Elective Year Could be Improved: Qualitative Results and Recommendations from Student Interviews

**DOI:** 10.3205/zma001161

**Published:** 2018-02-15

**Authors:** Samuel Beck, Christian Schirlo, Jan Breckwoldt

**Affiliations:** 1 University of Zurich, Faculty of Medicine, Dean's Office, Zurich, Switzerland

**Keywords:** clinical clerkship, undergraduate training, learning in workplace, situated learning, clinical teaching

## Abstract

**Background: **Entering the Clinical Elective Year (CEY) is a challenging transition phase for undergraduate medical students. Students become members of a professional team, thereby taking over certain tasks, which are executed more or less independently. Factors which facilitate (or impede) this transition in the perception of students are not well described. We therefore wanted to explore, what students perceived to be helpful during the first phase of the CEY and possibly derive respective recommendations.

**Methods: **We conducted semi-structured interviews with 5th year medical students after they had completed the first two months of their CEY. Students were asked which problems they had faced and how they felt prepared for the CEY. Interviews were audio-recorded, transcribed, and analysed by qualitative content analysis.

**Results:** From 34 interviews, we included 28 into analysis. Overall, 24 students were satisfied or very satisfied with their start into the CEY. Satisfaction was expressed with respect to workplace experiences, learning progress, responsibilities and team integration. Especially, students appreciated if they were integrated as active members of the team, were given responsibility for certain units of work, and received well-structured formal teaching and supervision. Students had divergent opinions about the quality of teaching and supervision, about their own achievements, and the recognition they received. Students recommended improvements in respect to formal teaching and supervision by clinical supervisors, preparation of the CEY by university, and supporting structures in the hosting institution.

**Conclusion: **Students in this study were generally satisfied with the first two months of their CEY. Facilitating factors were active and responsible involvement into routine patient care, and high quality formal teaching and supervision. Findings may inform universities, teaching hospitals, and students how to better shape the first phase of the CEY.

## Zusammenfassung

**Hintergrund: **Der Eintritt in das Wahlstudienjahr (WSJ) ist eine herausfordernde Übergangsphase für Medizinstudierende. Die Studierenden werden zu Mitgliedern eines professionellen Teams und übernehmen dabei Aufgaben, die sie mehr oder weniger selbständig ausführen. Faktoren, die diesen Übergang in der Wahrnehmung der Studierenden erleichtern (bzw. erschweren) sind bisher nicht ausreichend untersucht. Ziel dieser Studie war es daher zu analysieren, wodurch die erste Phase des WSJ aus Sicht der Studierenden erleichtert wurde, mit dem Ziel entsprechende Empfehlungen daraus ableiten.

**Methoden: **Wir führten semistrukturierte Interviews mit Medizinstudierenden durch, welche die ersten beiden Monate des WSJ absolviert hatten. Die Studierenden wurden befragt zu den Problemen, denen sie begegnet sind und wie sie sich auf das WSJ vorbereitet gefühlt hatten. Die Interviews wurden auf Tonband aufgezeichnet, dann transkribiert und mittels qualitativer Inhaltsanalyse ausgewertet.

**Ergebnisse:** 28 von 34 Interviews wurden in die Analyse einbezogen. Insgesamt waren 24 Studierende zufrieden oder sehr zufrieden mit ihrem Start ins WSJ. Zufriedenheitsfaktoren waren „Erfahrungen am Arbeitsplatz“, „Lernfortschritt“, „Verantwortung“ und die „Integration ins Team“. Die Studierenden schätzten es insbesondere, wenn sie als aktive Mitglieder ins Team einbezogen waren, ihnen Verantwortung für bestimmte Arbeitsbereiche übertragen wurde und sie gut strukturierten Unterricht sowie adäquate Supervision erhielten. Die Studierenden hatten unterschiedliche Meinungen zur Unterrichtsqualität, zur Supervision und zur Anerkennung, die sie erhielten. Die Studierenden machten Verbesserungsvorschläge hinsichtlich des formalen Unterrichts, der Supervision, der Vorbereitung auf das WSJ von Seiten der Universität und der Unterstützung in der jeweiligen Ausbildungseinrichtung.

**Schlussfolgerung: **Die Studierenden waren grundsätzlich zufrieden mit den ersten zwei Monaten ihres WSJ. Unterstützend wirkten sich dabei folgende Faktoren aus: Aktive und verantwortungsvolle Mitwirkung in der Patientenbetreuung sowie qualitativ hochwertiger Unterricht bzw. Supervision. Die Studienergebnisse geben Hinweise zur verbesserten Gestaltung der ersten Phase des WSJ für Universitäten, Lehrspitäler und Studierende.

## Background

Acquiring clinical experience as member of a professional team is crucial for the learning progress of medical students [1], [2], [3], [4], [5]. Therefore, clinical placements are part of many undergraduate curricula to provide the opportunity to act within a professional team [6] and to regularly and deliberately practice in workplace [7]. However, the impact of clinical rotations may be compromised by insufficient teaching, inadequate supervision, poorly defined responsibilities and difficulties in interdisciplinary or interprofessional collaboration [8], [9], [10], [11], [12], [13]. Deeper knowledge of the factors facilitating (or impeding) the first phase of the CEY could help to better shape this transition for both, students and hospitals. International literature in this field mainly deals with postgraduate learners and shows neglect of the undergraduate situation: learning processes within the CEY have even been regarded as a “black box” [14]. However, those studies which have been conducted in the undergraduate field focus on students’ expectations, experiences and achievements over the whole period of the placement [10], [11], [14] and not primarily on factors which are supportive for the start into the CEY itself. To get more insight into this transition phase, we conducted semi-structured interviews with students who had just gone through the first two months of their CEY, where the interviewees had already gathered some field experience, whilst on the other hand not had adapted to full routine. We thereby aimed to avoid that relevant aspects of transition were masked by repression and compensation strategies [15]. With data from these interviews we aimed to derive recommendations, how students could be deployed more effectively and how hospital and students, could gain more from the CEY with the final aim to improve patient care. To our knowledge comparable data have not been published for Switzerland yet. 

## Methods

### Curricular setting 

In German speaking Switzerland, students enter medical school after 12 years of primary, secondary and high school and after successfully passing a national aptitude test. Medical studies take six years until graduation by passing a federal licensing examination. The first two years focus on basic sciences, physiology, anatomy, integrating basic clinical and communication skills. During years three and four clinical medicine is delivered in a systematic fashion, of which about one third is given as clinical courses and bedside teaching. Workplace experiences of longer duration are not common; the only mandatory placement is a one-month practical rotation in nursing (PRN) at 3 out of 4 medical schools (not mandatory at Zurich Medical School). The entire fifth year, the clinical elective year (CEY), is mainly taken in different clinical institutions, partly also in research or public health institutions. Students choose disciplines and institutions freely, each placement is individually contracted between the student and the institution. About 98% of students choose a minimum of 2 months in internal medicine and 92% in surgery [16]. A logbook guides students through their CEY, including scheduled workplace based assessment. Learning objectives focus on clinical and communication skills and the application of clinical knowledge [16] and are related to the Swiss Catalogue of Learning Objectives (SCLO). A financial compensation of 800-1200 Swiss francs per month (equalling US dollars) is provided. The final, sixth year takes up experiences from the CEY and prepares for the final licensing exam, which is entirely based on the SCLO [http://sclo.smifk.ch [retrieved 2016 Aug 10]].

#### Study design 

Interviews were conducted with students during their third month of the CEY. We limited the study to students of the University of Zurich, who were on a rotation in either surgery, or internal medicine in hospitals within a 30-km radius of Zurich. 

We designed a specific, semi-structured questionnaire according to “guided interview” theory [17], [18]. It was based on results of a survey conducted by a working group of 6^th^ year students, and revised by the research team (medical educators, curriculum designers and a trained postgraduate student). 

The questionnaire was piloted with three students in their CEY, leading to minor revisions. Specific questions at the beginning of the interview were posed for “overall satisfaction of the first two CEY months” and for the “greatest challenges during this period”. In the main interview section, participants were asked open questions to describe which factors they perceived to facilitate (or impede) the start into the CEY, and what had prepared best for CEY. They were also asked about their interprofessional (IP) experiences, working hours, hospital size, income and previous clinical experiences. A translation of the interview guideline is provided online (see attachment 1 , file 1). To ensure consistent data collection, a single interviewer (SB) followed a specific written guide with the opportunity to ask for more details at defined points. In addition to extensive literature studies and pilot interviewing there was no further specific training. The interviewer was not working at the respective hospital and was not a member of the teaching staff.

#### Interviews 

In October and November 2013 potential participants were contacted by e-mail explaining study background and interview setting. A book voucher of 30 Swiss Francs was offered in case of participation. Interviews were held at the respective hospitals in a separate and quiet room. Before starting, an explanatory text was read to the participants and they were informed that the transcripts were blinded for analysis.

#### Data analysis

After written consent interviews were audiotaped and transcribed using the software “f5” [https://www.audiotranskription.de [retrieved 2017 May 06]]. Transcripts were blinded for qualitative content analysis [19]. Six of 34 (18%) transcripts were independently analysed by five experts in medical education (see acknowledgement) in order to find main categories according to the question: “Which factors facilitate (or impede) a successful start into the CEY?” In a subsequent discussion, the group agreed on three main categories: “experience at work”, “preparation (of the CEY)”, and “framing conditions”. On this basis further subcategories were formed by thematic analysis by two of the authors (SB, JB). Comments were sorted into subcategories and classified as negative, neutral and positive.

General satisfaction was related to potentially confounding factors, such as students’ gender, medical discipline, hospital size, and PRN). 

From the resulting material we finally derived recommendations how to facilitate the start of the CEY from a student perspective. 

For coding, the program “MAXQDA 11” was used [http://www.maxqda.de/ [retrieved 2017 May 06]]. For quality control 10% of the interviews were re-coded ten months after the first coding. 

#### Statistics

Sample size consideration was based on the assumption, that qualitative saturation of information would be reached at 20-25 interviews. To be able to account for sub-groups and for safety reasons we aimed at 30 interviews. Comparative statistics were not performed due to the qualitative character of the study.

#### Data safety and ethics approval

No personal data of interviewees were recorded. The ethical committee of the Canton of Zurich approved the study design (KEK Nr. 97-2015).

## Results

Out of 92 eligible students who received an invitation e-mail, 42 responded. Since we aimed at only 30 interviews no further reminders were sent out to recruit additional participants. In 34 cases interviews could be performed. The first three of them were used as pilots to refine the interview guide. From the remaining 31 interviews three more had to be excluded because these students had started a new rotation within the past two weeks. Finally 28 interviews were used for analysis, from which 944 comments could be classified. Students mean age was 24.6 years (SD 3.3, range: 22-41), other characteristics are shown in table 1 (Tab. 1). Actual interviewing time was 25 to 35 minutes. When re-coding comments 10 months later a concordance to sub-categories of 70% was found. 

### Specific opening questions 

Overall satisfaction with the CEY was high. Nine of the 28 interviewees stated to be “very satisfied” (++), 15 were “satisfied” (+) and the remaining four students answered “neutral” (0) /see table 2 (Tab. 2)). No obvious correlations were seen in relation to gender or discipline.

Forty-nine important challenges were named, most often related to the new learning environment (n=15) and the new professional role (n=11). Further challenges addressed medical expertise (n=8; e.g. *“taking a patient history without having much time”*), collaboration (n=5; e.g. *“it costs me quite an effort to step up to some other professional”*) and communication (n=5; e.g.* “to break the ice at the beginning and face a patient”*). 

#### Comments from main interview section

From the main interview section 867 comments were sorted into the three main categories pre-defined by the researcher panel: “Experience at Work” (n=541), “Preparation” (n=254) and “Framing Conditions” (n=72). Further subcategories and classifications as negative, neutral and positive are shown in table 3 (Tab. 3), and are described in the following.

#### A. Experience at work (n=541)

We sorted the comments in this category into the subcategories “Team Member”, “Training” and “Self Perception”. Further subcategories are described in the specific sections.

##### A.1 Team member (n=207)

The subcategory ***“Interprofessional (IP) Collaboration”*** (n=118) included 54 positive comments, frequently related to learning from another occupational group. Typical comments were: “*from the nurses* [you learn], *how to take care of the patient. How cordial and empathic they are*.”. The great majority of interviewees indicated collaboration with all other occupational groups as helpful. 

Among negative comments (n=48) two themes prevailed: “Misunderstandings in IP collaboration” (n=17) such as incorrect prescriptions and “conflicts between nurses and doctors” in which the student was not personally involved (n=15).

Requests were made concerning IP collaboration (n=6): Students wanted to learn more about nursing and wished more IP interaction or joint breaks.

In the subcategory ***“Physician Team”*** (n=64) positive comments prevailed (n=40; negative n=17). Especially *“privilege of working together with physicians”* (n=17), *“good integration”* (n=5) and *“loyal support”* (n=9) were positive comments, as opposed to “*disrespectfulness of doctors”* and *“being the lowest in hierarchy”*.

The subcategory ***“Communication (unclassified)”*** included 25 comments, which were mainly coded negative: Miscommunication (*“the communication is not good enough”*), insufficient feedback culture, and poor communication style. Notably, four students spontaneously stated, that they had missed to introduce themselves or had done it too late.

##### A.2 Training (n=64)

We divided this subcategory into “Supervision” (informal teaching) and “Formal Teaching”. Within “***Supervision***” (n=21) twelve comments were negative, e.g.: *“sometimes you have the impression of just sitting around and no one is interested what you are doing”*, opposed to nine positive ones, as: “*she [the resident] really took her time and told me, that I was here for learning.”*

For ***“Formal Teaching”*** (n=43) we also found a nearly balanced distribution of negative and positive comments. *“A negative example was in the emergency room. Residents who never explain things, never show interesting patients to you, but as soon as [the patients] have to stay in the hospital you have to complete the history and physical status”*, was one of 19 negative comments. From 21 positive comments one typical quotation was: *“The physicians explain a lot, they’re not stressed and take their time whenever you ask them*”.

##### A.3 Self Perception (n=270)

In this subcategory we formed five further subcategories.

Within ***“Practical Involvement”*** (n=123) about half of the comments were positive e.g. *“I was allowed to suture a wound in the emergency room, which was really fun.”* For nine students application of knowledge into practice was *“a personal, especially positive experience”* and 13 comments stressed the “importance to work practically”. About 30% of the comments were negative, often referring to work load, or to orders which were not communicated clearly, or were assigned to the student without being perceived as useful. Also, negative comments were made about administrative work (n=8). 

Under the subcategory ***“Personal Benefit”*** (n=58) the majority of comments was positive (n=48). Twenty-eight of these referred to the amount students had already learned and ten comments highlighted the appreciation they received.

Corresponding negative comments (n=9) were made for *“lack of recognition and appreciation”*, and *“lack of learning effects”*.

In the subcategory ***“Responsibility”*** (n=39) also most comments were positive. Especially patient contact was denoted to be important: *“[a personal positive experience was] when I could take care of a patient in the emergency room autonomously. Yes, these were the best moments”*. Three out of the five negative comments referred to not having enough responsibility *“they do not give us enough trust […] my resident controls everything”*. 

Comments in the subcategory ***“Achievement”*** (n=34) were balanced between positive and negative. A comment as: *“I think, [the tasks] are adapted to my capabilities, I’m neither overburdened nor inadequately challenged”* was contrasted by: *“[the nurses] are expecting everything of you, if you can’t do it, you’re out”*.

Twenty-two comments were made in respect to ***“Identification with Physician’s Role”*** (n=22) dealing with professionalism, role models, and the personal future. An exemplary negative comment was: “*it was a bit shocking […] when I saw how doctors interact with patients”*, while a typical positive comments was: *“You’re getting into it… you’ve got a view from back stage”*.

#### B. Preparation (n=254)

In this main category 250 comments were assigned to three subcategories, four comments could not be classified.

##### Prior experiences at university (n=150)

Positive comments (n=68) were especially made because of the good practical training (n=10), lectures (n=5) and lecture documents (n=3) provided by university. Specifically, clinical tutorials at general practitioners’ offices were viewed as helpful (n=7).

Students also frequently mentioned their self-studies as useful for their start into the CEY (n=12):* “I really learned how to examine a patient from internet-tutorials*”, or *“I prepared for the [US exam] and I think that helped a lot”*. Also, specific “exam preparations” (n=8), and “OSCE preparations” (n=6) were regarded as helpful. Ten comments were classified in “[I was] well prepared for CEY”. In nine cases students could not answer how the university could improve preparation (*“I don’t know what they [university] could have done more”*), and 16 students stated, that they would not have needed more support.

From 73 negative comments, 20 were related to clinical courses at university, especially course quality was regarded as improvable *“the level of courses varied a lot, there should be a quality-control. Sometimes you don’t learn anything”*. Also, some interviewees found, that groups had been too large, e.g.: *“our clinical course gathered five students around one bed to look at one patient from distance”*.

We identified 53 comments about what could help for a better start into the CEY, which we divided into 3 subcategories. In “clinical skills” (n=27) we summarised student’s wishes for practical training, including more clinical courses, more skills training, and more case-based seminars. 

In respect to the assignment of roles students suggested *that “[our] university would declare precisely what our tasks are and what we should learn”*. Six comments specifically referred to prepare for IP collaboration.

##### Prior experiences non university (n=38)

Experiences outside the university curriculum were almost exclusively rated positive. Typical comments were: *“I already worked in a hospital during my studies [which prepared well]”*. Seven students saw benefits in completing a PRN during the first two years of studies: *“…because here [in the PRN] you could see into it, it helped to understand a bit of the other profession”*. 

Notably, eleven students commented on personal attributes. Being *“open-minded and friendly”, “motivated”, “super-friendly”* or *“to be ambitious”* was stated to be important for a good start into the CEY.

##### Prior information (n=62)

When preparing for CEY students frequently retrieved information from elder students (n=26), mostly in verbal exchange (n=18). *“… to ask them [advanced students] how they behaved [in their CEY] helped a lot”*, is one example. Only two students stated that this was not helpful. The official CEY info-presentation at university was rated positive (n=4), negative (n=7) or neutral (n=5). 

#### C. Framing Conditions (n=72)

##### Income (n=30)

From the perspective of monetary compensation thirteen comments were coded as neutral and thirteen as negative, e.g.* “we are underpaid”* whereas four students were satisfied or stated: *“I would rather get less money but more teaching”. *

##### Structure of Curriculum and Supervision ratio (n=21)

Fifteen comments related to the structure of the CEY curriculum, four of them positive and eleven negative (*“there is no curriculum or a minimal requirement, what we should learn”*).

About supervision ratios only six comments were made: *“good 1:1 supervision” contrasted with “we [students] rarely work together with residents”*.

##### Working time (n=10)

Interviewees made ten comments on working hours, positive ones as often as negatives. Typical quotations were *“14 hours in one night, that was too much”, or “[working time] is totally ok”*.

##### Size of Hospital (n=8)

Six participants found their hospital was too large *“…therefore [I had] not that much contact to the patients”*, *“wouldn’t start here again”*. Two interviewees commented that a small hospital was good for starting. 

The four students whose overall satisfaction was neutral, all worked in larger hospitals, three of them thought their hospital was too large. Three of them felt not useful and not well integrated, and all four students found, that they did not get enough recognition and received insufficient teaching (see also table 2 (Tab. 2)).

## Discussion

Analysis of the interviews produced rich and detailed information on how students perceived the first two months of their CEY and on facilitating factors for the first phase of the CEY. Students expressed their thoughts, expectations and needs well and during the interviews we had the strong impression that they liked to talk about their experiences. This is consistent with their general satisfaction. These rather positive findings may be explained by a number of different conditions if compared to other reports, which found less positive experiences of students [10], [11]. On the other hand some authors also reported positive experiences, such as more than 80% of students recommending their clerkships to fellow students [20], or 75% of students who “enjoyed [their] first few weeks” of a clerkship [21]. As stated above, our students chose their institutions themselves and were employed on the basis of individual working contracts with each institution. Together with the monetary compensation students may have identified stronger with the institution, which in turn may have contributed to self-efficacy. A second reason might be that the SCLO (in combination with CEY portfolios and the final licensing examination) offered a clear goal of the CEY, thus providing consistent constructive alignment [22]. Finally, curricula in Switzerland give fewer opportunities for workplace experiences prior to the CEY making transition into the CEY more clear-cut. We think, that the material provides an authentic picture of the students’ situation due to the close relation to their working experience and the specific suggestions students made. In the following we will discuss facilitating and impeding factors of satisfaction. It is important to keep in mind that these findings represent a specific student’s perspective. We grouped suggestions according to the institutions (or groups) involved: university, teaching hospitals, and students themselves.

### Preparation by university 

Our interviewees found clinical courses at university to be very helpful to prepare for their CEY. However, potential for improvement was stated for bedside teaching, which students suggested to be expanded, to begin earlier within the curriculum, and to be delivered with more structure and at smaller group size. As this may be obvious in the eyes of students, it still has to be discussed on the background of total student workload [23] and of faculty resources. Regarding a PRN during the first two years of studies students made exclusively positive comments, even before they had been asked for this rotation explicitly. Students reported that it helped to understand and facilitate IP collaboration. However, students who had completed a PRN did not report greater satisfaction with their CEY.

Our interviewees further suggested to introduce a specific preparation course at university, addressing organisation and administration of a ward, writing patient reports and IP collaboration. This could easily be integrated into curricula [24], preferably by involving students who already completed their CEY.

#### Suggestions for Teaching Hospitals 

Students clearly identified a number of facilitating factors within their teaching hospitals. Firstly, they highly valued clear structure in various fields, such as transparent organisational structures, clear assignments of tasks, or well-defined students’ roles within the team. This is well in line with educational literature which highlights the provision of clear structures [11], [25], [26]. By supporting team integration and clarifying communicational structures, information flow could be improved, resulting in improved patient care.

##### Give responsibilities to students 

An important issue in the view of students was to be given a certain (graded) amount of responsibility. They pointed out, that these tasks should be an integral part of patient care and should be a meaningful contribution to the work of the team. This would enhance self-efficacy of students while at the same time it could help to spread work load within the team. This would imply to entrust students to perform specific tasks, based on supervisors’ decisions. The recently introduced concept of Entrustable Professional Activities (EPAs) may serve as a valuable model to make the principle of transferring responsibility visible [27], [28]. As EPAs are to be incorporated into the national Swiss Catalogue of Learning Objectives [29], our students’ comments support the application of this concept.

Interestingly, students asked for responsibilities already during the first two months. It may be speculated, that this request would become more pronounced as the CEY progresses.

##### Provide high-quality feedback and recognition 

It is not surprising, that students found high-quality supervision and formal teaching helpful. This confirms broad findings from literature, where feedback is acknowledged as one of the most powerful teaching strategies [30] especially if adapted to the specific needs of learners [31]. Furthermore, recognition and appreciation of work appeared to be an important aspect for the interviewees (including financial compensation). Although this is a subjective perception by students, it is nonetheless worth to know for supervisors, since (at least for postgraduate training) perception of feedback has been reported to be underestimated by trainees and at the same time overestimated by supervisors [32]. 

#### Implications for Students

Importantly, students also reflected on their own contribution to learning success within their CEY, which in our eyes indicates a proactive attitude among the interviewees. Students pointed at favorable personal attributes, such as being open-minded, actively seeking feedback, and to regulate learning in a self-directed fashion. Literature confirms respective attributes to be conducive for learning [32], [33]. While these attributes may be self-evident, we still find it important to make them explicit as professional behavior, especially to more introverted students. 

Students should also be encouraged to thoroughly select teaching institutions in respect to curriculum structure and teaching quality (via website, advanced students, and student organizations). 

Regarding hospital size, satisfaction was higher in middle- to small-size hospitals (see table 4 (Tab. 4)). However, students should be made aware, that medical quality is also dependent on an adequate number of cases [34]. 

#### Recommendations for university, teaching hospitals and students

Based on frequent and unequivocal mentions, and on confirmation by corresponding negative comments we derived a number of statements on conditions and strategies, which facilitate the beginning of the CEY. From these, we formulated recommendations for university, teaching hospitals and students, from a student’s perspective (see table 4 (Tab. 4)). 

#### Limitations 

The following limitations of this study have to be discussed. First, the interviewed students represent a selection in respect to the medical specialties and the locations of teaching hospitals, as well as the students’ response to the study invitation. The culture in other medical specialties or in hospitals outside the area of Zurich may be different (and may explain the positive view of the students). Regarding the response rate to our first invitation, 46% is in no way representative: It may well be assumed, that only those students responded who had made positive experiences. However, the purpose of the study was to identify facilitating and impeding factors for the start into the CEY and not to draw a comprehensive picture of the CEY. In this respect the interviewees gave a balanced view of supportive factors and aspects which could be improved.

As a second point, the interviewed students expressed their opinions after the first two CEY months and it remains unknown, how their perceptions may have changed during the further CEY. As a further point it is possible, that a social desirability bias had been present. We tried to counteract this by choosing an interviewer ‘at eye-level’, who had just graduated from medical school and who was neither involved in the clinical work of the interviewees, nor in their teaching.

Whether the findings can be transferred to other groups of students remains open as we used a convenience sample from one university. However, our results provide a solid basis for further studies.

## Conclusions

Students in this study highly valued their workplace experience when entering the CEY. Main factors perceived to be facilitating for this phase of the CEY were (a) to be an active part of a professional team, (b) to be responsible for certain units of professional work, and (c) to receive high quality feedback, well-structured formal teaching and supervision. 

Our results suggest strategies to facilitate students’ first steps into clinical work at the levels of universities, hospitals and students.

Further research has to be done to generalise and confirm our results, in order to make students, hospitals and patients benefit the most from the CEY. 

## List of abbreviations used

- CEY: Clinical Elective Year
- EPA: Entrustable Professional Activity
- IP: interprofessional
- PRN: Practical Rotation in Nursing

## Declarations

**Ethics:** Approval by the Ethical Committee of the Canton of Zurich (KEK Nr. 97-2015)

**Consent to publish: **All authors read and approved the final manuscript.

**Authors' contributions: **SB, Master of Medicine, Physician and doctoral student, co-designed the study, collected and processed the data, and prepared a draft of the manuscript. CS, physician, MD, MME, contributed to the study design, the interpretation of data, and gave intellectual input to the final version of the manuscript. JB, physician, MD, MME, designed the study (in collaboration with SB), contributed to organisational issues, and wrote the main parts of the manuscript.

**Acknowledgement **Sylvia Kaap-Fröhlich, Dr. rer. nat., MPH, Ernst Jünger, MD, Lorenzo Käser, MD, and Jutta Bisaz took their precious time to find consent on the main coding categories

**Availability of data and materials:** anonymised transcripts of interviews and coding schemes are available on request from the author.

**Funding/Support: **none (academic study)

## Competing interests

The authors declare that they have no competing interests. 

## Supplementary Material

Interview guidelines

## Figures and Tables

**Table 1:**
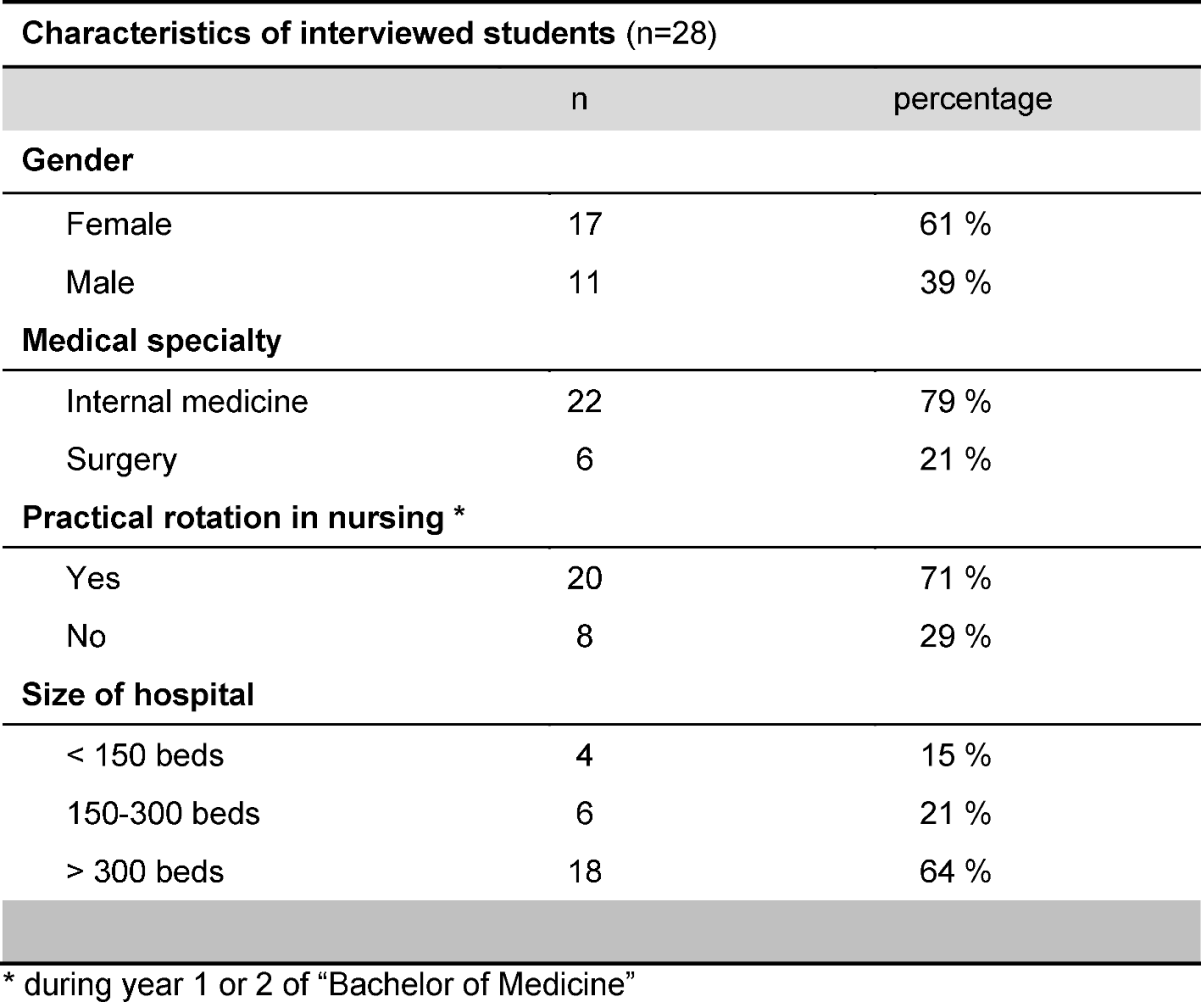
Characteristics of interviewed students

**Table 2:**
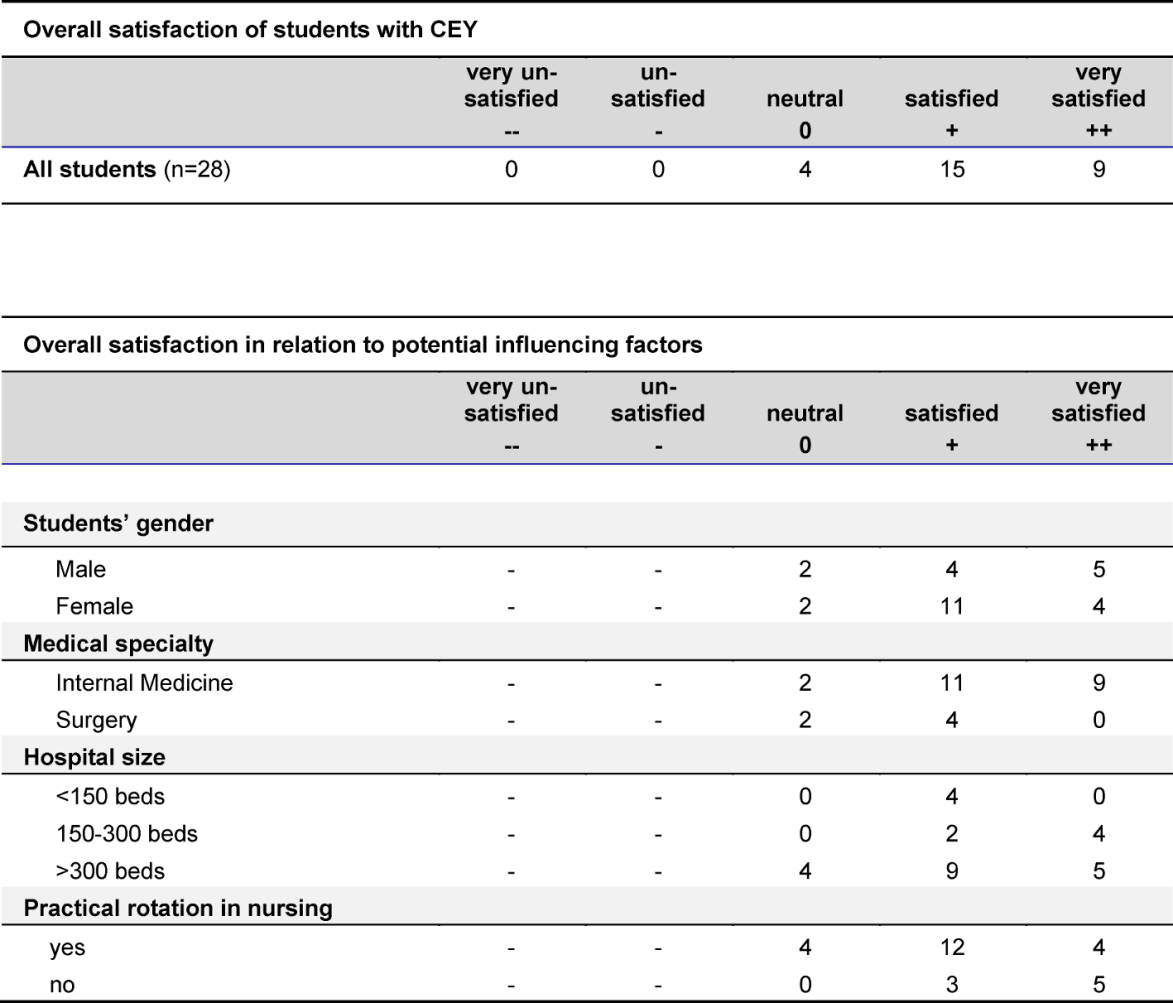
Students’ satisfaction with the first two months of their CEY (upper part) and (lower part:) overall satisfaction in relation to students’ gender, discipline, hospital size, and whether a practical rotation in nursing had been completed within the first two years of studies. (n=28 students)

**Table 3:**
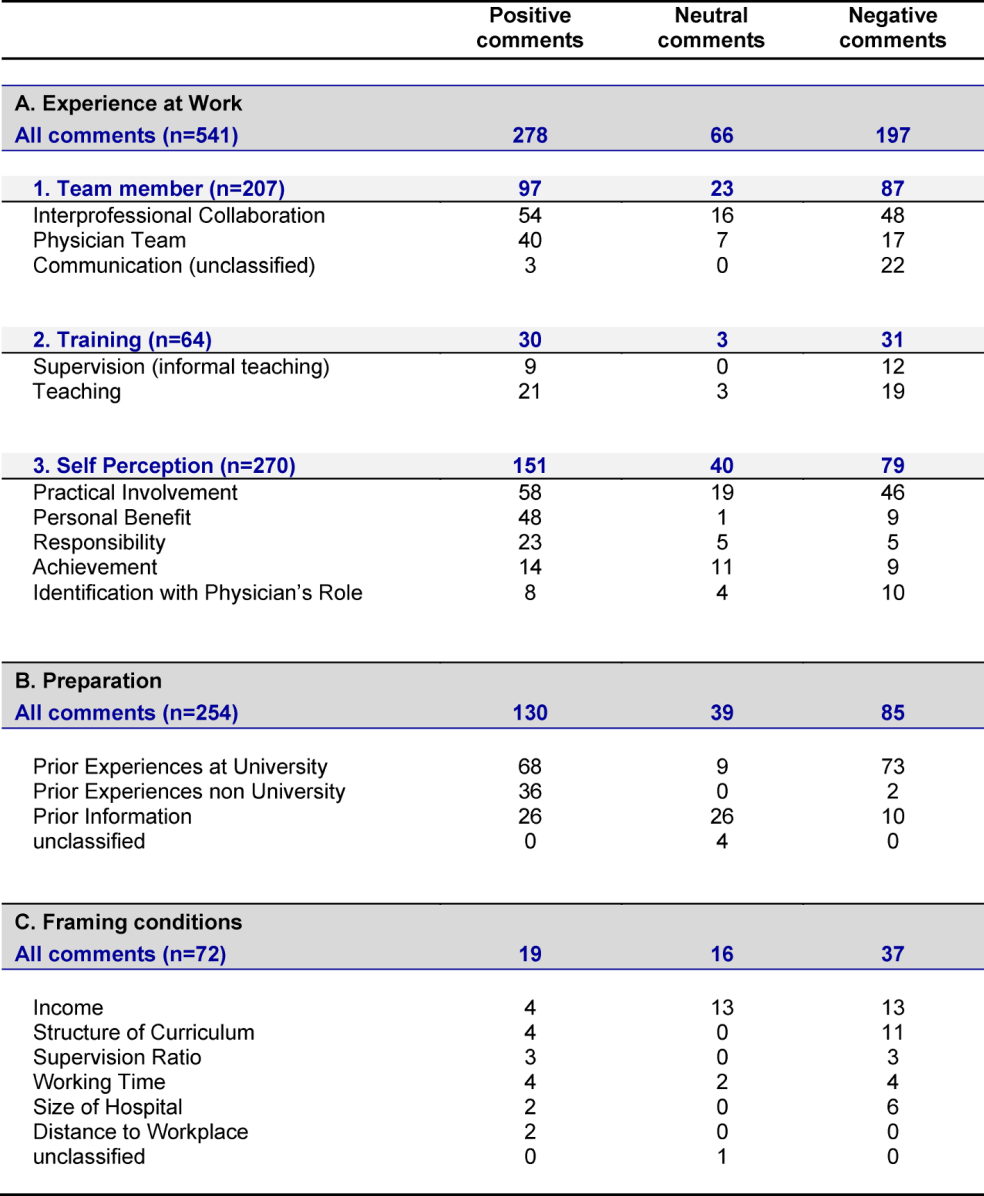
867 comments from 28 semi-structured student interviews, sorted into main categories (A-C), and subcategories

**Table 4:**
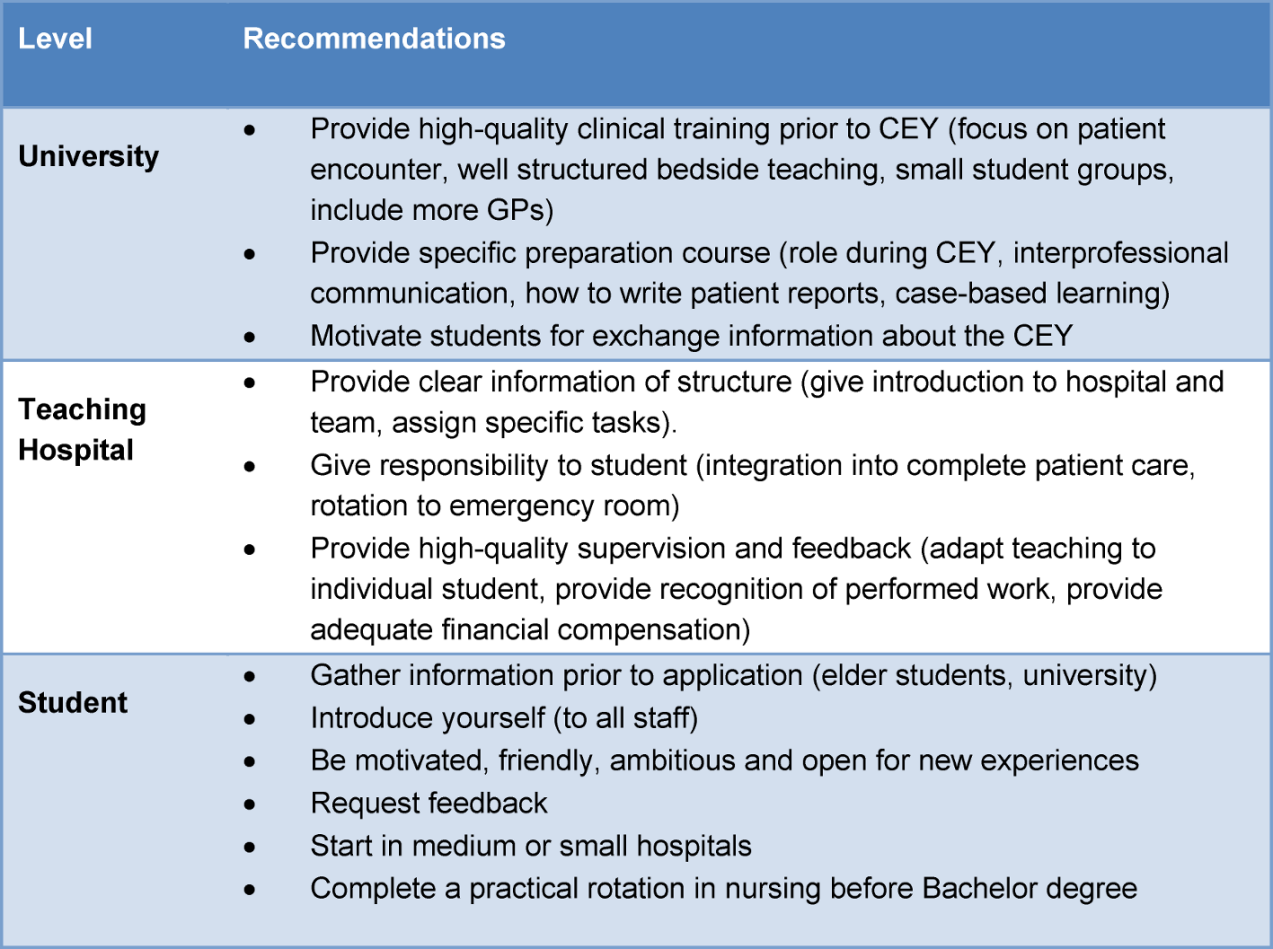
Recommendations to facilitate the start of the Clinical Elective Year, based on 28 semi-structured student interviews, University of Zurich
