# Supplementary material for: How the Start into the Clinical Elective Year Could be Improved: Qualitative Results and Recommendations from Student Interviews
Source: GMS J Med Educ. 2018 Feb 15;35(1):Doc14. doi: 10.3205/zma001161 (PMC5827187; doi:10.3205/zma001161)
Supplement: Interview guidelines [file JME-35-14-s-001.pdf]

Interview guideline/ questionnaire

- PRN *= Practical Rotation in Nursing*
- CEY *= Clinical Elective Year*
- italic type= information not for the participant
- normal= reading to the interviewee

Introduction:
„I'm going to read a text to you to ensure, that all students will get the same information.
Dear student
Thank you for participating in this study.
The interview will be divided into different parts. First, there will be a conversation based on a semi-structured guided interview. Our conversation will be recorded to make a transcript and perform coding afterwards. In this first part you should tell us your thoughts about a question or problem. There's no right or wrong.

In the second part, you should fill in a questionnaire. We will then ask you some further questions, but here you have to choose the answer that fits best. At the end we ask you to answer some questions about your curriculum. *****

Should you have any problems with understanding or comprehension, please ask immediately. You should answer honestly and on the basis of your own experiences.

All data will be anonymized before using them for written work, while particular quotations might be published. We are going to send a copy of this work to all participants.
The aim of this work is to collect experiences of the clinical elective year. Thanks to them the curriculum might be adapted.
The interview will last about 35 Minutes.
Thank you again for participating.

***** *this part of the interview was not included into the study due to a low number of participants in the subgroup without a PRN history.*

qualitative part approx. 20'
semi structured interview, recording for transcript and subsequent coding. All questions need to be asked.

Questions based on: conversation with the focus group of the "Fachverein" (educational committee), with a students' initiative within the 6th year of studies.

Information in advance

- *The interview should reflect the actual situation in the clinical elective year, 2-3 months after starting*
- *The questions should be referenced to this situation (otherwise mentioned specifically)*
- ***PRN** = Practical Rotation in Nursing = internship of 4 weeks, completed before graduating with Bachelor degree*

"In this part you should answer the question/ problem referenced to your situation at the moment in the clinical elective year."
If the student is reflecting to long, the phrase in the right column can be read out (note in the transcript)

<u>Introduction in the CEY, interprofessional teamwork: 11'</u>

**Main question / starting sequence**	**Proceeding question**
I'm satisfied with the CEY so far.	-- / - / 0 / + / ++
Could you specify this answer?	
What helped you beforehand to start easily into the CEY?	Info presentation at university? Reports of elder students?
Where did you acquire this knowledge and skills?	Self-learning (pre studies, leisure time, conversations)? Or regular studying ?
Would you have wished more support from your university?	Do you have specific suggestions?
What amazed you most (in the CEY)?	Was everything like expected?

Please tell me about a personal, especially positive experience in the CEY?	
Please tell me about a personal, especially negative experience in the CEY?	
How would you describe the income (money and learning effect)	
What responsibilities could you take over?	examples
Interprofessional questions	
List at least 4 occupation groups and specify briefly the function of those jobs.	
According to your experience - are there any problems between the different professions?	- could you describe those problems?<br>- how would you be better prepared to those problems?
Which occupational group could you profit the most from?	
Have you ever made a mistake in an interprofessional situation?	

General information about the test person: 5'
To be filled in by the interviewer. Follow-up questions to be asked according to the respective answer.

Question	Answer 1	Answer 2	Follow-up-question
Gender	M	W	
Age (in years)			
Medical studies = first higher education studies?	Y	N	
Did you complete every term of the medical studies in Zurich?	Y	N	
Did/Do you have a sideline job associated with medicine?	Y	N	What? How long (in weeks; 42h/week)?
Did/Do you have a sideline job not associated with medicine?	Y	N	What? How long (in weeks; 42h/week)?
Did you complete a practical rotation in nursing?	Y	N	Did you do it because you thought it was mandatory? Where, for how long?
Family members working in medical professions?	Y: father/ mother/ partner/ sibling working as a doctor	Y: father/ mother/ partner/ sibling working in health care, but not as a doctor	N
Clinical elective year: how many months have you already completed?			What disciplines?
Is German your mother tongue?	Y	N	If not: For how long have you been living in a German-speaking area?
General comments/ feedback to the questionnaire			

Questions PRN (if completed), maybe 3rd part beforehand (to know whether PRN was done or not) 4'

In what way has your PRN been useful for you?	- What advantages did you get from your PRN in respect to your clinical elective year?<br>- Were your expectations regarding the CEY realistic, as anticipated because of your PRN?<br>- Was your PRN useful regarding interprofessional work?
Tell me about a personal positive experience from your PRN	
Tell me about a personal negative experience from your PRN	
Have your expectations regarding the PRN been fulfilled?	In what way have they not been fulfilled ? (could you give examples?)
How has your decision to study medicine been influenced by your experiences from the PRN?	
What responsible jobs could you take over?	
Would you recommend medical students to absolve a PRN at the beginning of their studies?	
What advantages did you have because of the PRN compared to students who did not complete one?	
What disadvantages did you have because of the PRN compared to students who did not complete one?	
How could the PRN be made more attractive?	- What do you think about a catalogue of learning objectives?<br>- What would you think about a mandatory PRN?<br>- What would you think about replacing a "Mantelstudium" module by a mandatory PRN?

Questions PRN (if not completed), 4’

In what way could a PRN have been useful for you?	- What advantages could you have got from your PRN in respect to your CEY?<br><br>- Would your PRN have been useful regarding interprofessional work?
What should have been done for making you complete a PRN?	
Would your decision to study medicine have been influenced by your experiences from the PRN?	
Would you recommend medical students to do a PRN at the beginning of their studies?	
What advantages did you have because of not completing the PRN compared to students who completed one?	
What disadvantages did you have because of not doing a PRN compared to students who completed one?	
How could the PRN be made more attractive?	- What do you think about a catalogue of learning objectives?<br>- What would you think about a mandatory PRN?<br>- What would you think about replacing a “Mantelstudium” module by a mandatory PRN?
